# The histone deacetylase Hdac1 regulates inflammatory signalling in intestinal epithelial cells

**DOI:** 10.1186/s12950-014-0043-2

**Published:** 2014-12-20

**Authors:** Alexis Gonneaud, Julie Moore Gagné, Naomie Turgeon, Claude Asselin

**Affiliations:** Département d’anatomie et biologie cellulaire, Faculté de médecine et des sciences de la santé, Pavillon de recherche appliquée sur le cancer, Université de Sherbrooke, Sherbrooke, Québec J1E 4K8 Canada

**Keywords:** Hdac1, Inflammation, Intestinal epithelial cell, Chemokine expression

## Abstract

**Background:**

It has recently been found that both nuclear epithelial-expressed histone deacetylases Hdac1 and Hdac2 are important to insure intestinal homeostasis and control the mucosal inflammatory response *in vivo*. In addition, HDAC inhibitors modulate epithelial cell inflammatory responses in cancer cells. However, little is known of the specific role of different HDAC, notably Hdac1, in the regulation of inflammatory gene expression in intestinal epithelial cells (IEC).

**Methods:**

We investigated the role of Hdac1 in non-transformed IEC-6 rat cells infected with lentiviral vectors expressing specific Hdac1 shRNAs, to suppress Hdac1 expression. Proliferation was assessed by cell counting. Deacetylase activity was measured with a colorimetric HDAC assay. Cells were treated with IL-1β and/or the JQ1 bromodomain acetyl-binding inhibitor. Nuclear protein levels of Hdac1, Hdac2, phosphorylated or unphosphorylated NF-κB p65 or C/EBPβ, and NF-κB p50 and actin were determined by Western blot. Chemokine and acute phase protein expression was assessed by semi-quantitative RT-PCR analysis. Secreted cytokine and chemokine levels were assessed with a protein array. Chromatin immunoprecipitation experiments were done to assess RNA polymerase II recruitment.

**Results:**

Reduced Hdac1 protein levels led to Hdac2 protein increases and decreased cell proliferation. Hdac1 depletion prolonged nuclear IL-1β-induced phosphorylation of NF-κB p65 protein on Ser536 as opposed to total p65, and of C/EBPβ on Ser105. In addition, semi-quantitative RT-PCR analysis revealed three patterns of expression caused by Hdac1 depletion, namely increased basal and IL-1β-stimulated levels (Hp, Kng1), increased IL-1β-stimulated levels (Cxcl2) and decreased basal levels with normal IL-1β induction levels (Ccl2, Ccl5, Cxcl1, C3). Secreted cytokine and chemokine measurements confirmed that Hdac1 played roles both as an IL-1β signalling repressor and activator. Hdac1 depletion did not alter the JQ1 dependent inhibition of basal and IL-1β-induced inflammatory gene expression. Hdac1 depletion led to decreased basal levels of RNA polymerase II enrichment on the Ccl2 promoter, as opposed to the Gapdh promoter, correlating with decreased Ccl2 basal mRNA expression.

**Conclusions:**

Hdac1 is a major nuclear HDAC controlling IL-1β-dependent inflammatory response in IEC, notably by regulating gene-specific transcriptional responses. Hdac1 may be important in restricting basal and inflammatory-induced gene levels to defined ranges of expression.

**Electronic supplementary material:**

The online version of this article (doi:10.1186/s12950-014-0043-2) contains supplementary material, which is available to authorized users.

## Background

The intestinal epithelium plays important roles as a physical and biochemical sensor of the luminal environment [1]. Indeed, intestinal epithelial cells (IEC) regulate gut homeostasis by sensing luminal bacterial products or by responding to inflammatory signals emanating from the mucosal immune system [2,3]. In turn, these inflammatory signals regulate an IEC-specific inflammatory response, characterized by the expression of various cytokines, chemokines and acute phase proteins [4]. In recent years, epigenetic modifications, which include DNA methylation, histone methylation and acetylation among others, have been shown to act as receivers and transmitters of environmental changes, leading to variations in gene expression. Indeed, it has been suggested that many inflammatory diseases, including inflammatory bowel diseases, are affected by epigenetic modifications [5,6]. One important modification, namely lysine acetylation, is regulated by histone acetyltransferases and by histone deacetylases (HDAC), which respectively add or remove the acetyl group on histones as well as non-histone proteins [7]. Histone acetylation plays a dual role, either by reducing histone-DNA interactions, thereby creating an open chromatin configuration, or by acting as an anchor recognized by bromodomain-containing chromatin modifiers [8]. Some of these recruited proteins, such as Brd family members, play important roles in the regulation of transcriptional elongation as well as inflammation. HDAC-dependent regulation of protein acetylation levels leads to cell- and gene-specific transcriptional repression or activation [8].

Of the eighteen HDAC, class I Hdac1 gains access to chromatin as a homodimer or heterodimer with Hdac2, in Sin3, CoREST and NuRD multiprotein complexes [9,10]. Hdac1 is considered as a positive regulator of cell proliferation as Hdac1 depletion in mice results in growth deficiencies, correlating with increased expression of the p21 cyclin-dependent inhibitor [11,12]. In contrast to single gene deletion, tissue-specific dual deletion of Hdac1 and Hdac2 leads to homeostatic phenotypes, such as epidermal differentiation defects when deleted in epidermal cells [13], and intestinal homeostatic perturbations when deleted in intestinal epithelial cells [14]. Treating colon cancer cell lines with HDAC inhibitors or reducing Hdac1 expression suppresses colon cancer cell proliferation [15], and alters inflammatory signalling [16]. Hdac1 is also considered as a negative regulator of transcription factors involved in inflammatory responses. For example, Hdac1 deacetylates the p65 NF-κB subunit, leading to reduced transcriptional activity during inflammatory responses [17-19]. Both phosphorylation and acetylation modifications interact to insure full NF-κB transcriptional activity [18,20,21]. Likewise, C/EBPβ acetylation leads to positive or negative interactions with co-regulators, including HDAC [22].

HDAC inhibitors are being considered as pharmacological agents to modulate inflammatory responses. However, many studies have revealed opposite effects of HDAC inhibitors as suppressor or stimulator of inflammatory responses and gene expression [23]. These differences may result from the use of different cell lines or mouse models, suggesting cell-specific effects, or of HDAC inhibitors with different selectivity to the various HDAC isoforms, suggesting target-specific effects. As more specific HDAC inhibitors are being generated, it is thus of importance to assess the role of specific HDAC in distinct cell types critical to the regulation of inflammation, including IEC.

Little is known of the role of specific HDAC in the control of IEC inflammatory responses. In order to understand the role of Hdac1 in the IEC inflammatory response, we have used the non-transformed intestinal epithelial cell line IEC-6 to avoid the increased sensitivity to HDAC inhibition found in cancer cells. We had previously observed that Hdac1 silencing increased both basal and IL-1β stimulated mRNA expression of the acute phase protein gene haptoglobin [24]. We show here that Hdac1 regulates inflammatory gene expression in a gene-specific manner, and may display both repressive as well as activating actions. Hdac1-dependent regulation of IL-1β-mediated IEC inflammatory response may depend, in part, on the duration of the inflammatory signals, through prolonged nuclear maintenance of phosphorylated NF-κB p65 and C/EBPβ. In addition, Hdac1 may be implicated in the maintenance of chromatin stability and of proper expression ranges in response to inflammatory signals, for a subset of chemokine genes.

## Methods

### Cell culture

The 18- to 24-day-old rat small intestinal epithelial non-transformed cell line IEC-6 exhibits an undifferentiated small intestinal crypt cell phenotype and a normal karyotype (CRL-1592, ATCC, Manassas, VA) [25]. Confluent cells, grown in Dulbecco’s modified Eagle medium (DMEM) with 5% fetal bovine serum (FBS), were induced with or without 10 ng/ml of recombinant human IL-1β (Bioshop Canada, Burlington, ON, Canada) and 1 μM of the JQ1 bromodomain acetyl-binding inhibitor (Cayman Chemical Company, Ann Arbor, MI, USA) [26] for different times.

### Retroviral infection

80% confluent IEC-6 cells were infected with lentiviral particles in medium supplemented with 4 μg/ml polybrene (Sigma-Aldrich Canada, Oakville, ON, Canada), as we have done before [24,27]. Two days after infection, transfected cells were selected with 2 μg/ml puromycin (Sigma-Aldrich Canada, Oakville, ON). In addition to the short hairpin RNA (shRNA) lentiviral control (SHC002V, Sigma-Aldrich, St-Louis, MO, USA), the MISSION shRNA lentiviral Hdac1 **(**TRCN0000039402, Sigma-Aldrich, St-Louis, MO, USA**)**, whose sequence is conserved in rat, mouse and human, was selected. Two clones of each infection were expanded, and the shCtrl5, shCtrl9, shHDAC1-18 and shHDAC1-21 cell lines were further characterized for Hdac1 and Hdac2 expression.

### Cell growth measurement

Ten thousand shCtrl and shHDAC1 IEC-6 cells were plated in each well. Cell growth was measured between 3 and 9 days with a hemocytometer to determine the doubling time, as we have done before [28]. Each experiment was done three times in triplicate. Results, representative of two independent experiments, were analysed by unpaired *t* tests and were considered statistically significant with p ≤ 0.05 (GraphPad Prism 5, GraphPad Software, San Diego, CA, USA).

### Deacetylase activity measurement

125 μg of nuclear proteins prepared either from combined shCtrl IEC-6 cell populations or from combined shHDAC1 IEC-6 cell populations, were incubated overnight at 4°C, with protein A/G agarose complex (Santa Cruz Biotechnology, Santa Cruz, CA, USA) and antibodies against Hdac1 (New England Biolabs, Mississauga, ON, Canada), Hdac2 (Abcam Inc., Cambridge, MA, USA) or mSin3A (Santa Cruz Biotechnology, Santa Cruz, CA, USA). Immune complexes centrifuged and washed three times were used to measure deacetylase activity with a colorimetric HDAC assay kit (Active Motif North America, Carlsbad, CA, USA), following the manufacturer’s protocol. Optical density (OD) was determined on a microplate reader at 405 nm, and deacetylase activity was measured in pmoles/min/mg. Results are representative of two independent experiments.

### Western blot analysis

Cell nuclei, prepared as described previously [28], were resuspended in Laemmli buffer (62.5 mM Tris–HCl, pH 6.9, 2% SDS, 1% β-mercaptoethanol, 10% glycerol, and 0.04% bromophenol blue) to obtain nuclear protein extracts. To measure C/EBPβ and NF-κB p65 phosphorylation, confluent shCtrl and shHDAC1 IEC-6 cells were induced with 10 ng/ml of IL-1β for 10 min, 30 min, 1 h, 2 h and 4 h. Protein concentrations were measured by the Bradford method (Bio-Rad Protein Assay kit, Bio-Rad Laboratories, Mississauga, ON, Canada) or BCA method (Pierce BCA Protein Assay Kit, Thermo Scientific, Rockford, IL, USA). Proteins were loaded on a 10% SDS-polyacrylamide gel and electroblotted on a PVDF membrane (Roche Molecular Biochemicals, Laval, QC, Canada). Membranes were incubated 1 h at room temperature, with rabbit, mouse or goat polyclonal antibodies against Hdac1 and Hdac2 (Abcam Inc., Cambridge, MA, USA); phospho-C/EBPβ (Ser105), p65 and phospho-NF-κB p65 (Ser536) (Cell Signalling Technology Inc., Danvers, MA, USA); NF-κB p50 (Assay Designs, Ann Arbor, MI, USA); C/EBPβ and lamin B (Santa Cruz Biotechnology, Santa Cruz, CA, USA), as done before [24,27]. Immune complexes were revealed with Amersham ECL^TM^ Western blotting detection reagents (GE Healthcare, Buckinghamshire, UK), according to the manufacturer’s instructions. Results are representative of two to four independent experiments.

### Semi-quantitative RT-PCR analysis

ShCtrl and shHDAC1 IEC-6 cells were induced with or without 10 ng/ml IL-1β for 24 h, or pre-incubated without or with JQ1 inhibitor for 4 h, before induction with IL-1β for an additional 8 h. Total RNAs were isolated with the RNeasy Plus Mini kit (Qiagen, Mississauga, ON, Canada). cDNAs were synthesized from 1 μg of RNA, with oligo(dT_15_) and Superscript II reverse transcriptase (Invitrogen Life Technologies, Burlington, ON, Canada), following the manufacturer’s protocol. cDNA products were amplified with the Taq PCR Master Mix Kit (Qiagen, Mississauga, ON, Canada) with PCR primers designed against the corresponding *Rattus norvegicus* cDNAs for *Hdac1*, *Hdac2*, *Hdac3*, *Hdac8*, as well as selected IL-1β targets induced in IEC-6 cells, namely haptoglobin (*Hp*), thiostatin (*Kng1*), *Ccl2*, *Ccl5*, *Cxcl1*, *C3* and *Cxcl2*, and a non-induced target, namely *Cxcl12*. Oligonucleotides were chosen in order to generate products of approximately 500 bp (Additional file 1, http://bioinfo.ut.ee/primer3-0.4.0/primer3/). cDNA amplification was performed by a first 94°C cycle for 5 min, followed by 28 cycles of 1 min at 94°C, 45 sec starting at 62°C and decreasing in increments of 0.3°C every cycle, 1 min at 72°C, and a final cycle of 1 min at 94°C and 10 min at 72°C. Relative quantification was estimated by glyceraldehyde-3 phosphate-dehydrogenase (Gapdh) amplification. Amplified PCR products were separated on a 1.4% agarose gel and visualized by ethidium bromide staining. Results are representative of two independent experiments.

### Secreted cytokine and chemokine measurements

ShCtrl and shHDAC1 IEC-6 cells were cultured in a 300 μl volume in 24-well plates, with or without 10 ng/ml of IL-1β for 24 h. Control and shHDAC cell supernatants were combined, and levels of 19 cytokines/chemokines were measured with a RayBio Rat Cytokine Antibody Array (AAR-CYT-G1-8, RayBiotech Inc., Norcross, GA, USA), following the manufacturer’s protocol. Fluorescence intensity from two independent experiments was measured with a ScanArray Express Microarray Scanner (PerkinElmer Life and Analytical Sciences, Downers Grove, IL). Proteins measured include Ccl2, Ccl20, Cxcl2, Cxcl3, Cxcl5, Cx3cl1, IL-1α, IL-1β, IL-4, IL-6, IL-10, β-NGF,CNTF, GM-CSF, VEGF, IFN-γ, TNF-α, Timp1 and Lep.

### Chromatin immunoprecipitation

Chromatin immunoprecipitation assays were done as described by Svotelis *et al.* [29]. 10^7^ shCtrl and shHDAC1 IEC-6 cells were recovered and crosslinked in medium containing 1% formaldehyde for 10 min at room temperature, before stopping the reaction with glycine. Chromatin from lysed cells was sonicated with a BRANSON digital sonifier (model S-250D, Branson Ultrasonics, Danbury, CT, USA), for six cycles of 10 sec (shCtrl cells) and five cycles of 10 sec (shHDAC1 cells) at 15% sonication intensity, to obtain DNA fragments between 300 and 500 bp. Chromatin immunoprecipitation was performed with an antibody against hypo- and hyperphosphorylated forms of RNA polymerase II (CTDH48) (EMD Millipore, Billerica, MA, USA) and with protein A/G PLUS-agarose reagent pre-blocked with BSA (Santa Cruz Biotechnology, Santa Cruz, CA, USA). The input DNA, used to determine the amount of DNA for each immunoprecipitation, represents one percent of the lysate. Immunoprecipitated DNA was diluted 1:10 before semi-quantitative PCR amplification of 70 to 145 bp Ccl2 and Gapdh promoter and gene exon 2 downstream sequences (Additional file 2), for 30 or 32 cycles with the touchdown amplification protocol used for chemokine expression analysis. Results are representative of three independent experiments.

## Results

### Hdac1 depletion reduces IEC-6 cell proliferation

**Figure 1:**
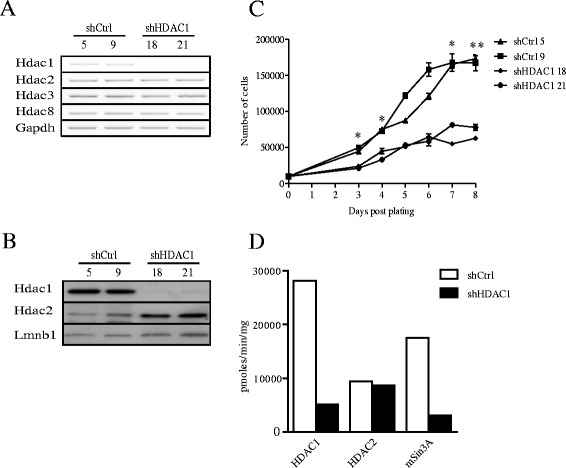
**Hdac1 depletion alters IEC-6 cell proliferation and deacetylase activity. A.** Total RNAs were extracted from two shCtrl (5; 9) or shHDAC1 (18; 21) IEC-6 cell lines. Class I Hdac1, Hdac2, Hdac3 and Hdac8 mRNA levels were measured by semi-quantitative RT-PCR, with Gapdh as a control for the amount of cDNA amplified. Results are representative of two independent experiments. **B**. Nuclear protein extracts from shCtrl (5; 9) and shHDAC1 (18; 21) IEC-6 cells were subjected to Western blot analysis, with Hdac1 and Hdac2 specific antibodies. Lamin B (Lmnb1) detection was used as a control for protein loading. **C**. 10,000 shCtrl (5; 9) and shHDAC1 (18; 21) IEC-6 cells were plated in each well of a 24-well plate. Cell growth was measured by cell counting with a hemocytometer between 3 and 8 days after seeding. Results are representative of two independent experiments. Differences in cell numbers between shCtrl and shHDAC1 cells are statistically significant (^*^ p ≤ 0.05; ^**^ p ≤ 0.01). ▲, shCtrl 5; ■, shCtrl 9; ♦, shHDAC1 18; ●, shHDAC1 21. **D**. Nuclear proteins from shCtrl and shHDAC1 IEC-6 cells were immunoprecipitated with specific antibodies against Hdac1, Hdac2 and mSin3a. Deacetylase activity was measured using a commercial colorimetric HDAC assay kit. Deacetylase activity was calculated in pmoles/min/mg. Results are representative of two independent experiments.

To determine the role of the histone deacetylase Hdac1 in IEC, we infected IEC-6 cells with a retroviral construct expressing a selected shRNA against Hdac1. We chose IEC-6 cells because, while being immortal, these cells are not tumorigenic and display a normal karyotype. Hdac1 mRNA and protein expression was reduced in both shHDAC1 cell lines, as determined respectively by semi-quantitative RT-PCR (Figure 1A) and Western blotting (Figure 1B). mRNA levels of other class I HDACs, namely Hdac2, Hdac3 and Hdac8, were not significantly altered (Figure 1A). However, while Hdac2 mRNA levels were not affected, Hdac2 protein levels were increased (Figure 1B). This post-transcriptional increase in Hdac2 protein levels after Hdac1 depletion was also observed in other cell types [11]. This was confirmed by immunofluorescence staining in shCtrl and shHDAC1 IEC-6 cell lines (data not shown).

Growth of Hdac1-depleted cells was reduced about 1.9-fold. Indeed, doubling times for both shHDAC1 cell lines were respectively 38 ± 3.1 h and 43.5 ± 3.7 h, as opposed to 22.2 ± 0.6 h and 20.7 ± 1.4 h for the shCtrl cell lines (p ≤ 0.05). In addition, cell number measurement showed a 50% reduced cell density for shHDAC1 cell lines, as compared to control cells (Figure 1C). Hdac1-depleted cells appeared larger, thinner, less cylindrical and refractive, when observed by phase contrast microscopy (data not shown). Thus, Hdac1 depletion decreases IEC-6 cell proliferation and modifies cell morphology.

### Hdac1 depletion leads to decreased deacetylase activity

Hdac1 and Hdac2 are subunits of Sin3A, NuRD and CoREST co-repressor complexes [7]. To determine whether Hdac1 depletion modulated the deacetylase activity of Hdac1-containing complexes, we immunoprecipitated nuclear Hdac1-, Hdac2- or Sin3A-containing complexes, and measured associated deacetylase activity. Deacetylase activity of Hdac2-containing complexes was not modified in the absence of Hdac1 (Figure 1D). In contrast, Hdac1- as well as Sin3A-containing complexes displayed reduced deacetylase activity in Hdac1-depleted cells. Of note, Hdac1 depletion did not significantly alter levels of co-repressor complex interacting proteins, such as Sin3A, Ing2, Mbd3, RbAp48, CoREST and Lsd1 (data not shown). Thus, Hdac1 depletion leads to decreased deacetylase activity associated with co-repressor complexes.

### Hdac1 depletion leads to prolonged nuclear localization of IL-1β-induced NF-κB p65 and C/EBPβ phosphorylated forms

**Figure 2:**
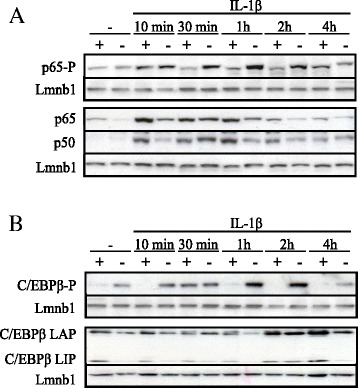
**Hdac1 depletion leads to prolonged nuclear NF-κB p65 and C/EBPβ phosphorylation after IL-1β induction in IEC-6 cells.** ShCtrl 5 (+) and shHDAC1 18 (−) IEC-6 cells were induced with 10 ng/ml of IL-1β for 10 min, 30 min, 1 h, 2 h and 4 h. Nuclear proteins were separated by 10% SDS-PAGE and proteins were analysed by Western blotting using specific antibodies against phosphorylated Ser536 or non-phosphorylated NF-κB p65 as well as against total NF-κB p50 **(A)**, or against phosphorylated Ser105 or non-phosphorylated C/EBPβ (LAP and LIP isoforms) **(B)**. Lamin B (Lmnb1) detection was used as a control for protein loading. Results are representative of four independent experiments.

We have previously shown that Hdac1 negatively regulated IL-1β-dependent induction of the acute phase protein haptoglobin in IEC-6 cells [24]. In order to uncover the impact of Hdac1 depletion on IL-1β signalling in IEC, we analysed the phosphorylation and expression levels of IL-1β signaling transcription factor targets, namely NF-κB p65 and p50 subunits as well as C/EBPβ. Basal nuclear p65 protein levels were reduced in Hdac1-depleted cells, and p50 levels were minimal (Figure 2A). In both shCtrl and shHDAC1 cells, p65 and p50 translocated to the nucleus after 10 min of IL-1β treatment, with a gradual decrease thereafter. We then verified the status of p65 Ser536 phosphorylation associated with increased transcriptional activation [18]. Basal nuclear p65 phosphorylated levels were increased in Hdac1-depleted cells. Interestingly, the time course of phosphorylation was different. Indeed, p65 phosphorylation occurred after 10 min of IL-1β treatment, in both shCtrl and shHDAC1 cell lines. However, whereas p65 phosphorylation decreased rapidly in control cells, p65 phosphorylation was maintained for more than 2 h in Hdac1-depleted cells (Figure 2A). These results show that Hdac1 depletion leads to increases in the proportion of nuclear phosphorylated p65, as opposed to total p65, at late times of IL-1β induction, and to different phosphorylation kinetics, suggesting prolonged activity. Of note, IL-1β did not modify Hdac1 and Hdac2 protein as well as mRNA expression in control and Hdac1-depleted cells (data not shown).

Regarding C/EBPβ, basal nuclear C/EBPβ LAP and LIP isoform levels were decreased in Hdac1-depleted cells (Figure 2B). In response to IL-1β, nuclear C/EBPβ protein levels increased after 2 h in control and Hdac1-depleted cells. We then verified C/EBPβ LAP Ser105 phosphorylation associated with increased transactivation [22]. Basal nuclear C/EBPβ LAP phosphorylated levels were increased in Hdac1-depleted cells, as opposed to control cells (Figure 2B). C/EBPβ phosphorylated levels transiently increased in control cells, with a peak at 30 min, after IL-1β induction. In contrast, C/EBPβ phosphorylated levels gradually increased for 2 h before a reduction at 4 h in Hdac1-depleted cells. Again, as for NF-κB p65, these results show that Hdac1 depletion leads to an increase in the proportion of phosphorylated C/EBPβ, as opposed to total C/EBPβ, and to different phosphorylation kinetics.

### Hdac1 differently modulates IL-1β-induced inflammatory response gene expression and cytokine secretion

**Figure 3:**
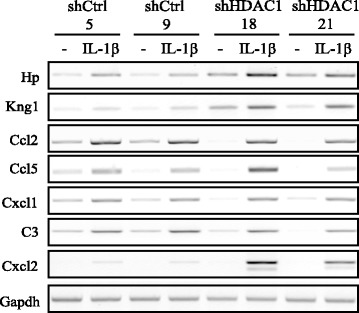
**Hdac1 depletion differentially modulates the expression of inflammatory response genes in IEC-6 cells.** Two shCtrl (5; 9) and shHDAC1 (18; 21) IEC-6 cell lines were treated with or without 10 ng/ml of IL-1β for 24 h. Total RNAs were isolated and expression levels of various inflammatory response genes (Hp, Kng1, Ccl2, Ccl5, Cxcl1, C3 and Cxcl2) were assessed by semi-quantitative RT-PCR. Gapdh was used as a control for the amount of cDNA amplified. Results are representative of two independent experiments.

We then analysed the expression of pro-inflammatory cytokines and chemokines in control and Hdac1-depleted cells after 24 h IL-1β treatment. Semi-quantitative RT-PCR analysis showed different patterns of expression in Hdac1-depleted cell lines, as compared to control cell lines. First, basal as well as IL-1β-induced mRNA levels of two acute phase protein genes, namely Hp and Kng1, were both increased in Hdac1-depleted cells (Figure 3). A second group, with increased IL-1β-induced levels in Hdac1-depleted cells, as opposed to control cells, included Cxcl2. Interestingly, a third group of genes, including Ccl2, Ccl5, Cxcl1 and C3, showed a decrease in basal levels, followed by similar IL-1β induction to that of control cells (Figure 3).

**Figure 4:**
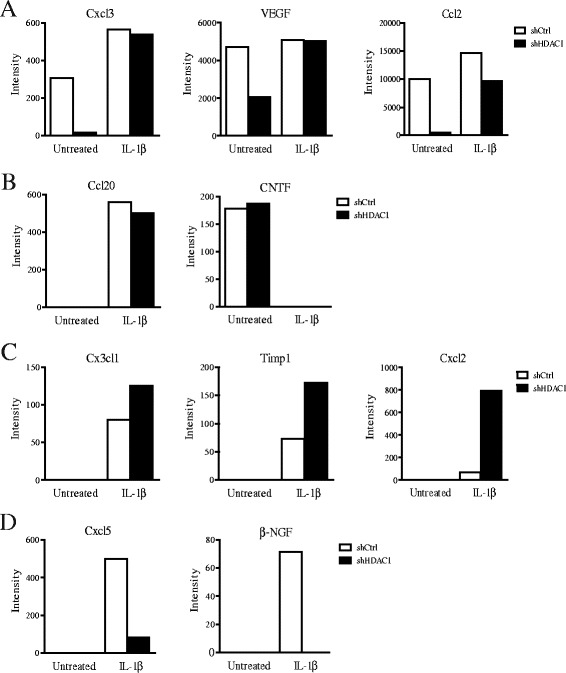
**Hdac1 depletion differentially modulates inflammatory protein secretion in IEC-6 cells.** Two shCtrl and shHDAC1 IEC-6 cells were treated with or without 10 ng/ml of IL-1β for 24 h. Supernatants from untreated or IL-1β treated shCtrl and shHDAC1 cells were harvested and cytokine secretion was measured with a commercial cytokine and chemokine array. The histograms represent the mean fluorescence intensity of duplicate experiments, measured with a ScanArray Express Microarray scanner. Secreted protein expression patterns are shown in **A** (Cxcl3, VEGF, Ccl2), **B** (Ccl20, CNTF), **C** (Cx3cl1, Timp1, Cxcl2), and **D** (Cxcl5, β-NGF).

To determine whether similar changes in inflammatory gene protein levels also occurred, we verified the expression of cytokines and chemokines with a protein array, in response to IL-1β. The same three patterns of expression were observed. First, Hdac1 depletion led to decreased basal protein expression of Cxcl3, Vegf and Ccl2, with similar levels of induction between control and Hdac1-depleted cells (Figure 4A). Ccl2 mRNA levels correlated with protein levels (Figure 3). Second, Ccl20 mRNA levels were increased and CNTF mRNA levels were decreased by IL-1β similarly in both cell types, suggesting a regulation independent of the presence or absence of Hdac1 (Figure 4B). Hdac1 depletion led to increased IL-1β-dependent induction of Cx3cl1, Timp1 and Cxcl2 protein levels (Figure 4C), without affecting basal protein levels. Cxcl2 mRNA levels correlated with protein levels (Figure 3). Finally, Cxcl5 and β-NGF IL-1β-induced levels were decreased in Hdac1-depleted cells (Figure 4D). These results uncover a role for Hdac1 as a repressor and an activator of inflammatory gene expression in response to IL-1β. Intriguingly, Hdac1 depletion renders expression of a subset of inflammatory genes more inducible in response to IL-1β, when compared to basal expression levels.

### JQ1 bromodomain acetyl-binding inhibitor reduces chemokine mRNA expression in IEC-6 cells

**Figure 5:**
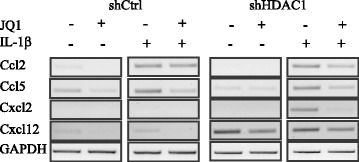
**Hdac1 depletion does not modify JQ1 inhibitory action on IL-1β-dependent Ccl2, Ccl5 and Cxcl2 mRNA expression in IEC-6 cells.** ShCtrl and shHDAC1 IEC-6 cells were pre-incubated for 4 h with 1 μM of the JQ1 bromodomain acetyl-binding inhibitor, before adding 10 ng/ml of IL-1β for an additional 8 h. Total RNAs were isolated and expression levels of various inflammatory response genes (Ccl2, Ccl5, Cxcl2 and Cxcl12) were assessed by semi-quantitative RT-PCR. ShCtrl and shHDAC1 cDNA fragments for Ccl2, Ccl5, Cxcl2, Cxcl12 and Gapdh were respectively separated by electrophoresis on the same gel, and were detected by ethidium bromide staining. Gapdh was used as a control for the amount of cDNA amplified. Results are representative of three independent experiments.

BET proteins interact with acetylated lysines on histones or transcription factors, through a BET domain [30]. BET protein Brd4 regulates transcriptional elongation of a subset of LPS-induced genes in macrophages [31]. Selective pharmacological inhibition of BET interaction with acetylated lysines, with I-BET [32] and JQ1 inhibitors [26,33,34], impairs inflammatory gene expression in macrophages, suggesting a critical and gene-specific role for BET proteins in the control of inflammation. To determine whether HDAC1 depletion affected BET protein dependent regulation of inflammatory gene expression in IEC, we treated non-induced and IL-1β-induced shCtrl and shHDAC1 cells with the JQ1 inhibitor. Basal levels of Ccl2, Ccl5, Cxcl2 and Cxcl12 mRNAs were reduced after JQ1 BET protein inhibitor addition, in control cells (Figure 5). Hdac1-depleted cells displayed reduced basal mRNA levels of Ccl2, Ccl5 and Cxcl2, while Cxcl12 mRNA levels were increased. JQ1 treatment did decrease both Ccl5 and Cxcl12 basal mRNA levels in shHDAC1 cells. Interestingly, JQ1 treatment resulted in similar decreased expression of IL-1β-induced chemokine genes in both control and Hdac1-depleted cells, suggesting that Hdac1 depletion does not alter the BET protein dependent regulation of chemokine genes in response to IL-1β.

### Hdac1 depletion reduces RNA polymerase II recruitment at the Ccl2 promoter in IEC-6 cells

**Figure 6:**
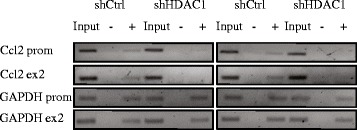
**Hdac1 depletion alters RNA polymerase II enrichment at the Ccl2 promoter in IEC-6 cells.** Chromatin immunoprecipitation assays were performed with chromatin extracts from shCtrl and shHDAC1 IEC-6 cells, without antibody (−), or with antibodies against RNA polymerase II (+). Input and immunoprecipitated samples were subjected to semi-quantitative PCR analysis with oligonucleotides amplifying Ccl2 and Gapdh promoter (prom) and downstream gene body (ex 2) sequences. Results from two independent experiments are shown.

Our previous results show that Hdac1 depletion leads to a reduction of basal mRNA levels of a subset of chemokines, namely Ccl2, Ccl5 and Cxcl1. To verify whether this decrease implicated RNA polymerase II promoter recruitment, RNA polymerase II elongation or both, we determined by chromatin immunoprecipitation, the recruitment of RNA polymerase II to Ccl2 promoter and exon 2 sequences, with the housekeeping gene Gapdh as a control. Our data show that RNA polymerase II was recruited to Gapdh promoter and exon 2 sequences in both shCtrl and shHDAC1 cells, suggesting that Hdac1 depletion does not affect RNA polymerase II engagement to the Gapdh gene (Figure 6). However, RNA polymerase II recruitment to both Ccl2 promoter and exon 2 regions was reduced in shHDAC1 cells, as opposed to shCtrl cells. Thus, Hdac1 depletion could result in basal gene-specific chromatin alterations which affect RNA polymerase II recruitment. Of interest, while these modifications alter basal expression levels of a subset of genes, such as Ccl2, IL-1β mediated transcriptional activation is still observed.

## Discussion

Our results establish a gene-specific role for Hdac1 in the control of IL-1β-dependent inflammatory response in IEC. We have shown that Hdac1 depletion leads to a decrease in Sin3a-associated deacetylase activity. While Hdac2 protein levels are increased, this does not result in Hdac2-associated increases in deacetylase activity. This suggests that Hdac2-dependent deacetylase activity is not as efficient as Hdac1-dependent activity. Indeed, Hdac2 deletion in embryonic stem cells, in contrast to Hdac1 deletion, does not significantly reduce Hdac activity of various co-repressor complexes [35]. In addition, Hdac2 depletion in IEC-6 cells does not alter cell proliferation or gene expression patterns of chemokines in response to IL-1β (data not shown). Thus, the more important contribution of Hdac1 deacetylase activity could explain, in part, the Hdac1-dependent regulation of inflammatory gene expression.

We show that Hdac1 depletion leads to increased nuclear maintenance of phosphorylated forms of two major regulators of the inflammatory response, namely NF-κB p65 and C/EBPβ, suggesting that Hdac1 may modulate the duration of inflammatory signals. Both phosphorylated forms are associated with increased transcriptional activation [18,22]. It has been shown that Hdac1 and Hdac3 deacetylate NF-κB p65, leading to decreased transcriptional activity [18]. In addition, it has been suggested that Hdac3-dependent NF-κB p65 deacetylation promotes a nuclear interaction between NF-κB p65 and the inhibitor protein IκBα, leading to NF-κB p65 nuclear export [20]. In contrast, our data show a similar time-dependent nuclear export pattern for NF-κB p65 and p50 in both control and Hdac1-depleted cells, while a selective maintenance of the phosphorylated and transcriptionally activated NF-κB p65 form is observed. This is not specific to NF-κB since we observe the same selective sustenance of a C/EBPβ phosphorylated form. Thus, Hdac1 may regulate the duration of the inflammatory response by modulating specifically the pattern of nuclear export of phosphorylated NF-κB p65 and C/EBPβ. Both NF-κB p65 and C/EBPβ are acetylated, and this acetylation stimulates transcription [18,22]. It is possible that increased nuclear levels of phosphorylation of both NF-κB p65 and C/EBPβ are enhanced and maintained by pre-existing increased acetylation modifications in Hdac1 depleted cells. Indeed, a positive interplay between S536 NF-κB p65 phosphorylation and acetylation for transcriptional activation has been proposed [20,21]. Thus, Hdac1 depletion may prolong inflammatory signals in IEC-6 cells, by insuring a selective time-dependent maintenance of NF-κB p65 and C/EBPβ activation. In addition to increased global histone acetylation resulting from Hdac1 depletion (Gonneaud et al., submitted), maintenance of inflammatory transcription factor activity may be involved in the increased expression of inflammatory genes, such as Hp and Cxcl2, in response to IL-1β in IEC-6 cells.

The pattern of expression of a subset of inflammatory genes, including Ccl2 and Ccl5, is intriguing. Indeed, while Hdac1 depletion leads to decreased basal mRNA levels, Hdac1 deficiency does not alter the IL-1β-dependent induction, since the same level of expression is achieved in shHDAC1 cells as in control cells. Thus, the end result is an enhanced range of expression in response to IL-1β when Hdac1 is depleted. This could in part be due to the maintenance of the nuclear inflammatory signals, as assessed by increased duration of nuclear phosphorylation of inflammatory transcription factors. Of note, both NF-κB and C/EBPβ are considered to be involved in the regulation of these genes.

Many inflammatory genes are characterized by the presence of paused RNA polymerase II complexes at their promoter [31]. Inflammatory stimuli then lead to transcriptional elongation induction. BRD proteins, such as Brd2 and Brd4, which bind acetylated residues on histones and transcription factors, are important regulators of transcriptional elongation of a subset of inflammatory genes with paused polymerase II [30]. We show here that IL-1β-dependent expression of some chemokine genes depends on BRD proteins in IEC, as assessed by pharmacological inhibition with the JQ1 bromodomain acetyl-binding inhibitor. Interestingly, Hdac1 deficiency does not modify the level of response to the inhibitor, suggesting that increased global acetylation levels do not alter BRD-dependent transcriptional processes after IL-1β stimulation. However, Hdac1 depletion leads to decreased basal mRNA levels of a subset of inflammatory genes. We show that diminished Ccl2 gene basal expression may be in part due to alterations in the recruitment of RNA polymerase II-containing complexes to promoter regions, as assessed by chromatin immunoprecipitation assays. Thus, Hdac1 may act as a co-activator insuring basal expression levels. Indeed, Hdac1 is required for the induction of a subset of glucocorticoid responsive genes [36]. In addition, transcriptomic studies of cells treated with HDAC inhibitors or selectively depleted in HDAC have identified both reduced and induced gene expression patterns, suggesting that the action of HDAC is not entirely repressive [37]. Chromatin immunoprecipitation studies have found that HDAC, including Hdac1, as well as HAT complexes, co-localize at active genes [38]. Based on these results, it has been proposed that HDAC may enable novel rounds of transcriptional initiation by clearing the promoter of newly deposited chromatin acetylated modifications. Hdac1 depletion may alter deacetylase activity of Hdac1-containing multiprotein co-repressor complexes [9], leading to increased chromatin acetylation and to defects in associated chromatin-modifying activities, such as chromatin remodelling. These modifications may destabilize the chromatin, leading to either repressive or activating gene-specific chromatin environment. For example, genes such as Ccl2, Ccl5, Cxcl1 and C3 may be more sensitive to these altered modifications, leading to decrease in basal expression levels and a more repressive chromatin.

## Conclusion

In conclusion, we have shown that Hdac1 is a major HDAC controlling the IEC IL-1β-dependent inflammatory response. Hdac1 may play important roles in regulating duration of IL-1β-mediated IEC inflammatory signals, gene-selective maintenance of chromatin stability and proper expression ranges in response to inflammatory signals.

## Additional files

Additional file 1:
**Oligonucleotides used for semi-quantitative RT-PCR analysis.**
Additional file 2:
**Oligonucleotides used for PCR chromatin immunoprecipitation analysis.**
