# Supplementary material for: The histone deacetylase Hdac1 regulates inflammatory signalling in intestinal epithelial cells
Source: J Inflamm (Lond). 2014 Dec 20;11:43. doi: 10.1186/s12950-014-0043-2 (PMC4299484; doi:10.1186/s12950-014-0043-2)
Supplement: Additional file 1: — Oligonucleotides used for semi-quantitative RT-PCR analysis. [file 12950_2014_43_MOESM1_ESM.docx]

| Additional file 1. Oligonucleotides used for semi-quantitative RT-PCR analysis | | | |
| --- | --- | --- | --- |
|  |  |  |  |
| *Gene* | *Region* | *Up* | *Down* |
|  |  |  |  |
| Hdac1 | 641 to 1225 | 5'-CTGGGGACCTACGGGATATT-'3 | 5'-TGTCAGGGTCTTCCTCATCC -'3 |
| Hdac2 | 568 to 1163 | 5'-TGAAGCTGAACCGTCAACAG-'3 | 5'-AAGGGCAACTGCAGTCTCAT -'3 |
| Hdac3 | 847-1415 | 5’-TGCTTCAATCTCAGCATTCG-‘3 | 5’-AGTTCAGACCTGGGAGAGCA-‘3 |
| Hdac8 | 570-1132 | 5’- CCAGAAGGTCAGCCAAGAAG-‘3 | 5’- CTGCTCCCAGCTGTAGAACC-‘3 |
| Hp | 634 to 1034 | 5'-CCTGCCTTCCAAAGACTACG-'3 | 5'-GGACCCAGTCCTTCAGATCA-'3 |
| Kng1 | 1080 to 1524 | 5'-AGGAGGCACTTGGTCATTCC-'3 | 5'-GAAGCTTCTGCCTGATGGTC-'3 |
| Ccl2 | 126 to 544 | 5'-CCAGAAACCAGCCAACTCTC-'3 | 5'-AGGCATCACATTCCAAATCA-'3 |
| Ccl5 | 176 to 396 | 5'-GTGCCCACGTGAAGGAGTAT-'3 | 5'-ATCCCCAGCTGGTTAGGACT-'3 |
| Cxcl1 | 291 to 833 | 5'-CCCCATGGTTCAGAAGATTG-'3 | 5'-AGGCATTGTGCCCTACAAAC-'3 |
| C3 | 2050 to 2546 | 5'-GCTCAGTGCAGTTGATGGAA-'3 | 5'-CAGTCGCAGGTCAATGAAGA-'3 |
| Cxcl2 | 445 to 849 | 5'-CCCTGCCTTACAGGAACAGA-'3 | 5'-ATACATTTCCCTGCCGTCAC-'3 |
| Cxcl12 | 179 to 257 | 5’-TGAAGCCAGTTGGGATAAGG-3’ | 5’-TAGGCAGGCTGTCTCCATCT-3’ |
| Gapdh | 3 to 499 | 5'-CCAAAGTTGTCATGGATGAC-'3 | 5'-GTGAAGGTCGGTGTGAACGG-'3 |
