# Supplementary material for: The histone deacetylase Hdac1 regulates inflammatory signalling in intestinal epithelial cells
Source: J Inflamm (Lond). 2014 Dec 20;11:43. doi: 10.1186/s12950-014-0043-2 (PMC4299484; doi:10.1186/s12950-014-0043-2)
Supplement: Additional file 2: — Oligonucleotides used for PCR chromatin immunoprecipitation analysis. [file 12950_2014_43_MOESM2_ESM.docx]

| Additional file 2. Oligonucleotides used for PCR chromatin immunoprecipitation analysis | | | |
| --- | --- | --- | --- |
|  |  |  |  |
| *Gene* | *Region* | *Up* | *Down* |
| Ccl2 prom | -96 to 45 | 5’-ACTCATCGAGGATGATGCTG-3’ | 5’-TGAGAGTTGGCTGGTTTCTG-3’ |
| Ccl2 ex2 | 831 to 926 | 5’-ATGCAGTTAATGCCCCACTC -3’ | 5’-TGCTGCTGGTGATTCTCTTG-3’ |
| Gapdh prom | -97 to -5 | 5’-TTGAGCTGGGACTGGATGAG -3’ | 5’-GGGCTGCAGTCCGTATTTATAG-3’ |
| Gapdh ex2 | 264 to 343 | 5’-ACGCTAATCTGACTTTCTTCTCC-3’ | 5’-CTGGAACTCACCCGTTCAC-3’ |
